# Strain variation in gene expression impact of hyphal cyclin Hgc1 in *Candida albicans*

**DOI:** 10.1093/g3journal/jkad151

**Published:** 2023-07-05

**Authors:** Anupam Sharma, Aaron P Mitchell

**Affiliations:** Department of Microbiology, University of Georgia, Athens, GA 30602, USA; Department of Microbiology, University of Georgia, Athens, GA 30602, USA

**Keywords:** *Candida albicans*, hyphal growth, strain variation, gene regulation

## Abstract

Formation of hyphae is a key virulence trait of the fungal pathogen *Candida albicans*. Hypha morphogenesis depends upon the cyclin Hgc1, which acts together with cyclin-dependent protein kinase Cdc28 to phosphorylate effectors that drive polarized growth. Hgc1 has also been implicated in gene regulation through its effects on 2 transcription factors, Efg1 and Ume6. Here, we report RNA-sequencing (RNA-seq) analysis of 2 pairs of *hgc1*Δ/Δ mutants and their respective wild-type strains, which lie in 2 different genetic backgrounds. We find that *hgc1*Δ/Δ mutations alter expression of 271 genes in both genetic backgrounds and 266 of those genes respond consistently with regard to up- or down-regulation. Consistency is similar to what has been observed with *efg1*Δ/Δ mutations and greater than observed with *nrg1*Δ/Δ mutations in these 2 backgrounds. The gene expression response includes genes under Efg1 control, as expected from prior studies. Hgc1-responsive genes also include ergosterol biosynthetic genes and bud neck-related genes, which may reflect interactions between Hgc1 and additional transcription factors as well as effects of Hgc1 on cellular length-to-width ratios.

## Introduction

*Candida albicans* is a commensal fungus that causes mucosal and lethal invasive infections in at-risk individuals (Polvi *et al*. 2015). One of the most well-studied virulence traits of this organism is its ability to transition from the yeast form to filamentous hyphae (Sudbery 2011). Hyphae are noteworthy both for their long tubular morphology and for their high-level expression of a set of hypha-associated genes (Mayer *et al*. 2013; Wilson *et al*. 2016; Chow *et al*. 2021). These genes specify multiple functions, including a panel of regulators that reinforce and modulate the hyphal gene expression program; an arsenal of virulence effectors that include adhesins and invasins, the Candidalysin toxin, diverse hydrolases, and neutrophil defense functions; and the hyphal-specific G1 cyclin 1 (*HGC1*) gene product, a hypha-specific cyclin (Mayer *et al*. 2013; Wang 2016; Wilson *et al*. 2016; Rodriguez *et al*. 2020).

*HGC1 is* expressed constitutively and independently of the cell cycle in hyphal cells (Zheng *et al*. 2004). Hgc1 partners with the cyclin-dependent kinase (CDK) Cdc28 to phosphorylate numerous effector proteins, thus promoting polarized growth at the hyphal tip (Zheng *et al*. 2004; Wang 2016; Chow *et al*. 2021). This role of Hgc1 has been extremely well established through phosphorylation assays, target site mutations, and other approaches (Wang 2016; Chow *et al*. 2021). However, a second role of Hgc1 in gene regulation is less well understood. Hgc1 has been shown to affect 2 transcription factors that promote hypha formation, Efg1 and Ume6. Efg1 is phosphorylated by the Hgc1-Cdc28 complex (Wang *et al*. 2009). Phosphorylation increases Efg1 binding to promoters of cell separation genes, such as *CHT3*, *SCW11*, and *DSE1*, that are activated by the transcription factor Ace2 (Wang *et al*. 2009). The relationship between Ume6 and Hgc1 is more complex, because Hgc1 has 2 characterized functions: to promote Ume6 degradation and to stimulate translation of the *UME6* mRNA (Mendelsohn *et al*. 2017). Therefore, Hgc1 affects the levels or activity of at least 2 transcriptional regulators.

Although Hgc1 affects Efg1 and Ume6, previous studies have detected little if any effect of an *hgc1*Δ/Δ mutation on the expression of hypha-associated genes (Zheng *et al*. 2007; Chao *et al*. 2010; Wang 2016; Chow *et al*. 2021). These studies used state-of-the-art methods at the time—Northern blot analysis and qRT-PCR—but were limited by sensitivity and the use of gene-targeted strategies. In addition, the studies addressed gene expression impact only in derivatives of the *C. albicans*-type strain SC5314. Reliance on a type strain has been vital for progress in our understanding of *C. albicans* (see for example Noble and Johnson 2007; Crunden and Diezmann 2021; Wang *et al*. 2021), but comparisons among strains can indicate which gene expression features have greatest functional significance (Morschhäuser *et al.* 2007; Huang *et al*. 2019; Wang *et al*. 2021; Do *et al*. 2022; Cravener *et al*. 2023). Here, we present genome-wide expression profiles of *hgc1*Δ/Δ mutants in 2 different *C. albicans* genetic backgrounds. Our findings indicate that the scope of Hgc1 gene expression impact is maintained in both strains, though the magnitude varies, in keeping with differences in phenotypic impact we recently reported (Sharma *et al*. 2023).

## Materials and methods

### Media

All strains used in the study are listed in Supplementary Table 1. They were maintained in 15% glycerol stocks stored at −80°C. Before all experiments, strains were grown on Yeast extract-Peptone-Dextrose (YPD) (2% Bacto Peptone, 2% dextrose, and 1% yeast extract) for 2 days at 30°C. For RNA-sequencing (RNA-seq) experiments, cells were grown in RPMI 1640 medium (Sigma-Aldrich), adjusted to pH 7.4 and supplemented with 10% fetal bovine serum (Atlanta Biologicals).

### RNA extraction and NanoString

RNA extractions and NanoString analysis were done according to the previously described method (Woolford *et al*. 2016). Briefly, RNA extraction was performed using a Qiagen RNeasy mini kit (Cat#74104) with some modifications. Then, samples were loaded on nCounter SPRINT cartridge for scanning on an nCounter digital analyzer. Raw counts were normalized against average total counts with background subtraction.

### RNA sequencing

RNA was extracted from the mentioned strains using a Qiagen RNeasy mini kit (Cat#74104) according to the previously described method with some modifications (Woolford *et al*. 2016). A total amount of 1-*μ*g RNA per sample was used as input material for the RNA-seq sample preparations. Sequencing libraries were generated using NEBNext Ultra RNA Library Prep Kit for Illumina (NEB, USA) following the manufacturer’s recommendations, and index codes were added to attribute sequences to each sample. To select cDNA fragments of preferentially 150–200 bp in length, the library fragments were purified with the AMPure XP system (Beckman Coulter, Beverly, USA). Then, 3-*μ*l USER Enzyme (NEB, USA) was used with size-selected, adaptor-ligated cDNA at 37°C for 15 min followed by 5 min at 95°C before PCR. Then, PCR was performed with Phusion High-Fidelity DNA polymerase, Universal PCR primers, and Index (X) Primer. At last, PCR products were purified (AMPure XP system), and library quality was assessed on the Agilent Bioanalyzer 2100 system. The clustering of the index-coded samples was performed on a cBot Cluster Generation System using PE Cluster Kit cBot-HS (Illumina) according to the manufacturer’s instructions. After cluster generation, the library preparations were sequenced on an Illumina platform, and 125-/150-bp paired-end reads were generated. The index of the *C. albicans* reference genome (Assembly A21) was built using hisat2 2.1.0, and paired-end clean reads were aligned to the reference genome using HISAT2. FeatureCounts v1.5.0-p3 was used to count the reads numbers mapped to each gene. And then, the Fragments Per Kilobase of transcript per Million mapped reads (FPKM) of each gene was calculated based on the length of the gene and the reads count mapped to this gene. Differential expression analysis between 2 conditions/groups (3 biological replicates per condition) was performed using the DESeq2 R package (1.14.1). Genes with an adjusted *P*-value < 0.05 found by DESeq2 were assigned as differentially expressed. Pearson correlations between replicates were in the range of 0.952–0.998, while Pearson correlations of WT vs mutant comparisons were in the range 0.921–0.986 (Supplementary File 2, “Sample correlations” tab).

### Software

Gene ontology (GO) term enrichment was performed using the FungiFun online tool. Venn diagram was made using online tool Draw Venn Diagram (ugent.be).

## Results and discussion

### Impact of Hgc1 on hypha-associated gene expression

We used NanoString assays for an initial determination of the effect of an *hgc1*Δ/Δ mutation on hypha-associated gene expression (Supplementary File 1). We included *hgc1*Δ/Δ mutants of 5 clinical isolates: SC5314 (clade 1), P76067 (clade 2), P57055 (clade 3), GC75 (clade 4), and 19F (clade 1). Cells were grown under strong hypha-inducing conditions (RPMI + serum at 37°C for 4 h). One hundred sixty genes were included in the assay, many of which are associated with hyphal growth and biofilm formation (Huang *et al*. 2019). The results suggested that several genes were differentially expressed in each mutant compared with its respective wild type, including hypha-associated genes *SAP5*, *HYR1*, *SAP6*, *HWP1*, and *ECE1* (Supplementary Fig. 1). More severe gene expression defects were observed in genetic backgrounds with more severe morphogenesis defects (Sharma *et al*. 2023). The results also suggested that the magnitude of gene expression differences between mutant and wild type may vary with strain background.

**Fig. 1.**
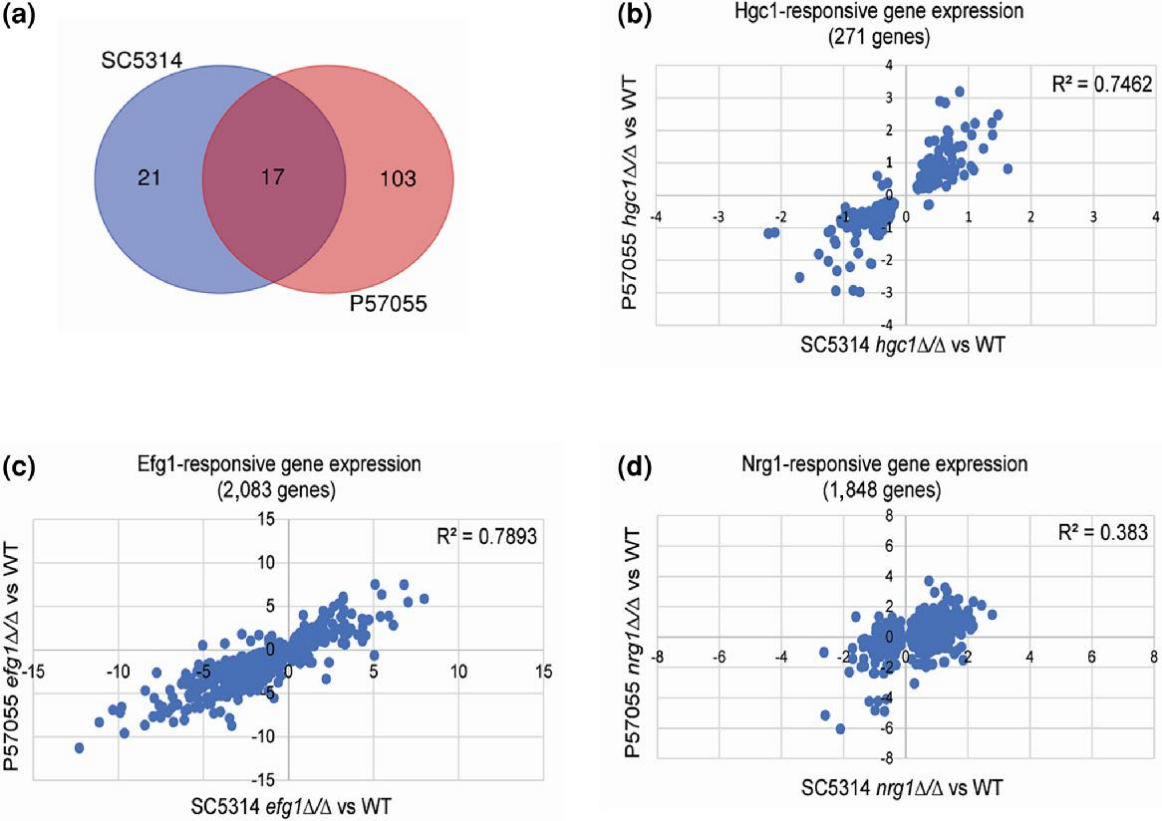
Gene expression analysis of *hgc1*Δ/Δ mutants. a) Global expression was assayed using RNA-seq. Three biological replicates were analyzed for *hgc1*Δ/Δ mutant and wild type of SC5314 and P57055 clinical isolates from cells grown in RPMI + 10% serum for 4 h at 37°C. Venn diagrams depict the genes dependent upon *HGC1* in both strain backgrounds with >2-fold expression change and *P* < 0.05. b) Regression analysis shows the number of significantly regulated (*P* < 0.05) *HGC1*-responsive genes, regardless of fold change. The *X* and *Y* axes represent *hgc1*Δ/Δ vs wild-type log fold change for SC5314 and P57055, respectively. The data sets correlate with *R*^2^ = 0.74. c) Regression analysis shows the number of significantly regulated (*P* < 0.05) *EFG1*-responsive genes, regardless of fold change, from previously published data (Cravener *et al*. 2023). The *X* and *Y* axes represent *efg1*Δ/Δ vs wild-type log fold change for SC5314 and P57055, respectively. The data sets correlate with *R*^2^ = 0.78. d) Regression analysis shows the number of significantly regulated (*P* < 0.05) *NRG1*-responsive genes, regardless of fold change, from previously published data (Mao *et al*. 2023). The *X* and *Y* axes represent *nrg1*Δ/Δ vs wild-type log fold change for SC5314 and P57055, respectively. The data sets correlate with *R*^2^ = 0.38.

We pursued these findings through RNA-seq analysis of the SC5314 and P57055 strain backgrounds after hyphal induction in RPMI + serum at 37°C for 4 h (Supplementary File 2). Gene expression impact of the *hgc1*Δ/Δ mutation varied considerably between the 2 backgrounds (Fig. 1a), based on conventional thresholds of an adjusted *P*-value < 0.05 and a fold change > 2 (Supplementary File 2, DEG tabs). In SC5314, 38 genes had altered expression in the *hgc1*Δ/Δ mutant. In P57055, 120 genes had altered expression in the *hgc1*Δ/Δ mutant. Only 17 genes responded significantly in both strain backgrounds based on these cutoffs (Fig. 1a). The outcomes mirrored previous findings regarding strain variation in the impact of transcription factor gene mutations (Morschhäuser *et al.* 2007; Huang *et al*. 2019; Do *et al*. 2022; Cravener *et al*. 2023; Mao *et al*. 2023).

**Fig. 2.**
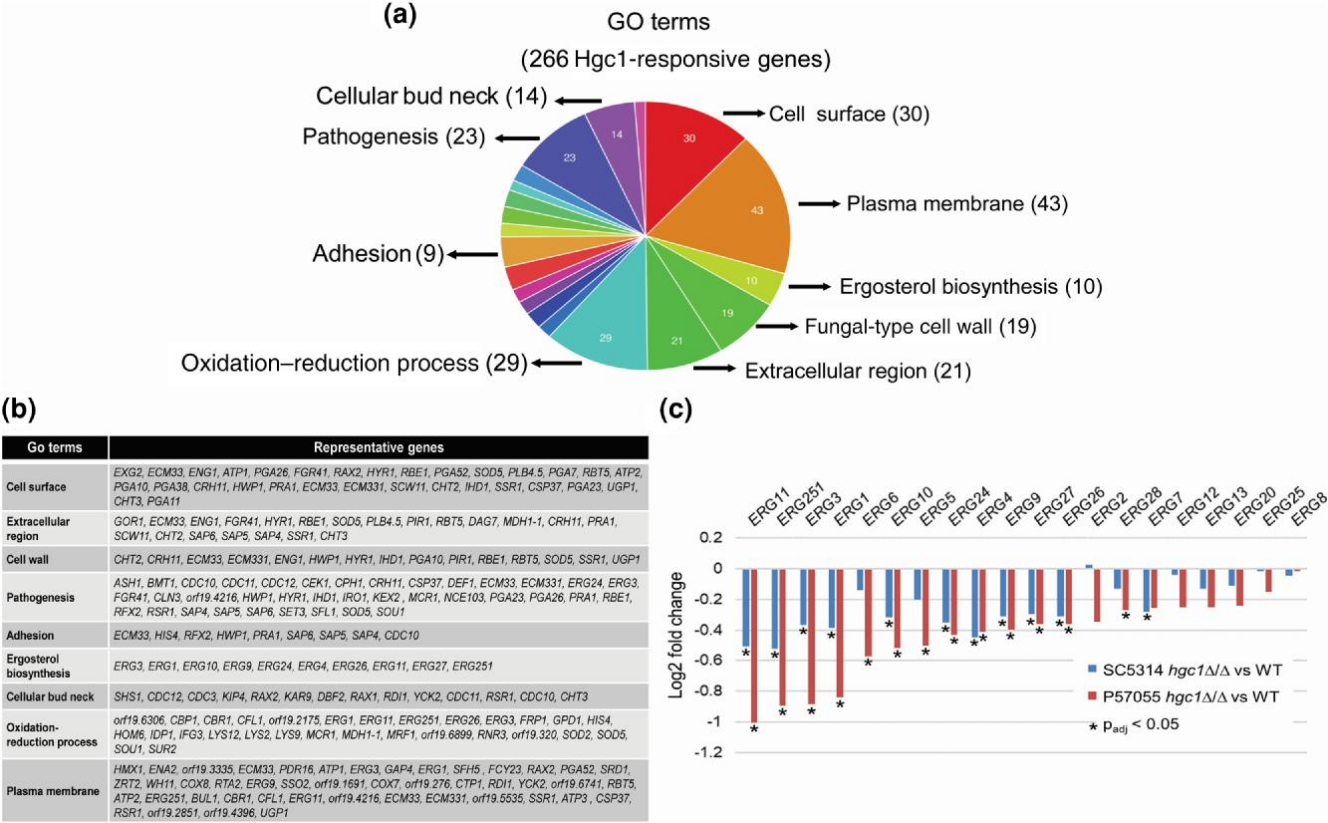
GO term analysis of Hgc1-regulated genes. a) The *HGC1*-responsive genes that were significantly regulated (*P* < 0.05) in *hgc1* mutants of SC5314 and P57055 background were analyzed for GO term enrichment using online tool FungiFun. Pie chart showing the major GO term distribution of the genes. b) Table showing the gene names representing significant GO terms. c) Graph of log_2_ fold change values for expression of *ERG* genes in *hgc1*Δ/Δ mutants vs WT of SC5314 (blue bars) and P57055 (red bars) backgrounds. An asterisk marks log fold-change values with a *P*_adj_ value of <0.05 (Supplementary File 2, “RNA SEQ LOG2 FOLD CHANGE” tab).

Many genes showed a similar trend in both strain backgrounds, but fold-change values were less in strain SC5314 than in strain P57055. This difference in gene expression may underly the more modest severity of the *hgc1*Δ/Δ morphogenesis defect in strain SC5314 than in strain P57055 (Sharma *et al*. 2023). To determine whether there may be underlying uniform impact of *hgc1*Δ/Δ mutations, we compared genes with statistically significant expression changes (adjusted *P*-value < 0.05) in the 2 strain backgrounds regardless of their fold change (Supplementary File 2, fold-change comparison tab). There were 271 genes in that group and 266 of the genes responded similarly (up-regulated or down-regulated) to *hgc1*Δ/Δ mutations in both strains. The results for the 2 strain backgrounds correlated with an *R*^2^ = 0.75 (Fig. 1b). The most significantly enriched GO terms for the genes with correlated responses included cell surface, plasma membrane, ergosterol biosynthesis, and cell wall (Fig. 2). These categories describe down-regulated genes for the most part (Fig. 2 and Supplementary File 3). Especially noteworthy was the down-regulation of many ergosterol biosynthetic genes in the *hgc1*Δ/Δ mutants of both strains (Fig. 2c). Overall, the effects of *hgc1*Δ/Δ mutations on gene expression align with the effects on hypha formation, in that the mutant defect is evident in both strains but less severe in SC5314 than in P57055 (Sharma *et al*. 2023).

How strong is the correlation between Hgc1-responsive genes when only statistical significance, not fold change, is used as a cutoff? To address this question, we examined data sets for 2 published mutant vs wild-type RNA-seq comparisons that were carried out with the same 2 strains backgrounds, SC5314 and P57055. One study examined *efg1*Δ/Δ mutants of both strains (Cravener *et al*. 2023). From those data sets, Efg1-responsive gene expression changes affected 2,083 genes and correlated with an *R*^2^ = 0.79 between the strains (Fig. 1c). A second study examined *nrg1*Δ/Δ mutants of both strains (Mao *et al*. 2023). From those data sets, Nrg1-responsive gene expression changes affected 1,848 genes and correlated with an *R*^2^ = 0.38 between the strains (Fig. 1c). Therefore, Hgc1-responsive gene expression changes correlate about as well as Efg1-responsive gene expression changes and considerably better that Nrg1-responsive gene expression changes.

How may Hgc1 influence gene expression? Work from Wang *et al*. (2009) showed that the Hgc1-Cdc28 complex phosphorylates transcription factor Efg1, causing it to antagonize the transcriptional activator Ace2. Their mutant analysis indicated that phospho-Efg1 represses Ace2-regulated cell separation genes, including *CHT3*, *SCW11*, *DSE1*, and *PGA38*. Three of these genes—*CHT3*, *SCW11*, and *PGA38*—are among the up-regulated genes that we found in *hgc1*Δ/Δ mutants. This relationship prompted us to make a broader comparison of Hgc1-responsive genes and Ace2 target genes. Among 271 Hgc1-responsive genes, we found 12 to 22 Ace2-regulated genes that had been identified in the studies of van Wijlick *et al*. (2016) and Desai *et al*. (2015). This comparison suggests that the impact of Hgc1 extends beyond the Ace2 regulatory circuit. Does Hgc1 act primarily through interaction with Efg1? Among 271 Hgc1-responsive genes identified, 113 genes have 5′ regions that are bound by Efg1 in multiple strains (Do *et al*. 2022); only 25 of the 113 genes show Efg1-dependent expression changes in multiple backgrounds (Cravener *et al*. 2023). Therefore, our results are not only consistent with the established role of Hgc1 in stimulating Efg1-DNA binding (Wang *et al*. 2009; Wang 2016) but also argue that Hgc1 affects gene expression independently of Efg1.

To our knowledge, the results presented here are the first genome-wide analysis of Hgc1-responsive gene expression. The effect of an *hgc1*Δ/Δ mutation on gene expression under our growth conditions is modest in magnitude but relatively consistent between 2 genetic backgrounds in scope. We note that we have used only 1 growth medium, temperature, and time point for gene expression assays, and expression differences may vary qualitatively or quantitatively under other conditions. Hgc1-responsive genes include some under Ace2 or Efg1 control, as expected from prior mechanistic studies. Hgc1-responsive genes also include some surprises, such as ergosterol biosynthetic genes and bud neck-related genes, that may reflect interactions between Hgc1 and additional transcription factors. Our findings help to illuminate the breadth of roles of Hgc1 in *C. albicans* biology.

## Supplementary Material

jkad151_Supplementary_Data

## Data Availability

All data necessary to support the conclusions of this study are available in this manuscript, its supplementary files, and our NCBI-deposited RNA-seq data sets with accession number GSE198594. Supplemental material available at G3 online.
