# Supplementary material for: Strain variation in gene expression impact of hyphal cyclin Hgc1 in Candida albicans
Source: G3 (Bethesda). 2023 Jul 5;13(9):jkad151. doi: 10.1093/g3journal/jkad151 (PMC10468301; doi:10.1093/g3journal/jkad151)
Supplement: jkad151_Supplementary_Data [file jkad151_supplementary_data.zip › Figure_S1_Legend_G3-2023-404262.docx]

**Fig S1. Nanostring gene expression analysis of the *hgc1∆/∆* mutants.** (**A)** Nanostring expression analysis was done on RNA extracted from cells grown in RPMI + 10% serum for 4 hr at 37°C. Hierarchal clustering of gene expression data was performed using MeV software. Fold change values were obtained by dividing normalized expression values for each *hgc1∆/∆* mutant by the wild-type strain for each of the probes. Upper (yellow) and lower bounds (blue) correspond to log2 fold change values of 2 and -2, respectively. **(B)** The graph shows the log2 fold change in the RNA levels of hypha-associated genes in the *hgc1∆/∆* mutants of five strain backgrounds.
