# Supplementary material for: Strain variation in gene expression impact of hyphal cyclin Hgc1 in Candida albicans
Source: G3 (Bethesda). 2023 Jul 5;13(9):jkad151. doi: 10.1093/g3journal/jkad151 (PMC10468301; doi:10.1093/g3journal/jkad151)
Supplement: jkad151_Supplementary_Data [file jkad151_supplementary_data.zip › Table_S1_G3-2023-404262.docx]

Sharma et al. Supplementary information

Supplementary Table S1

| **Strain no.** | **Strain** | **Species** | **Genotype/Parent/Source** |
| --- | --- | --- | --- |
| ASM1 | SC5314 | *C.albicans* | Wild type clinical isolate |
| ASM5 | P76067 | *C.albicans* | Wild type clinical isolate |
| ASM3 | P57055 | *C.albicans* | Wild type clinical isolate |
| ASM7 | GC75 | *C.albicans* | Wild type clinical isolate |
| ASM9 | 19F | *C.albicans* | Wild type clinical isolate |
| ASM35 | SC5314 *hgc1∆/∆* | *C.albicans* | Sharma et al., 2023 |
| ASM27 | P76067 *hgc1∆/∆* | *C.albicans* | Sharma et al., 2023 |
| ASM33 | P57055 *hgc1∆/∆* | *C.albicans* | Sharma et al., 2023 |
| ASM120 | GC75 *hgc1∆/∆* | *C.albicans* | Sharma et al., 2023 |
| ASM118 | 19F *hgc1∆/∆* | *C.albicans* | Sharma et al., 2023 |

**Supplementary Table S1: *Candida albicans* strains used in this study**
