# Supplementary figures and images for: Strain variation in gene expression impact of hyphal cyclin Hgc1 in Candida albicans
Source: G3 (Bethesda). 2023 Jul 5;13(9):jkad151. doi: 10.1093/g3journal/jkad151 (PMC10468301; doi:10.1093/g3journal/jkad151)

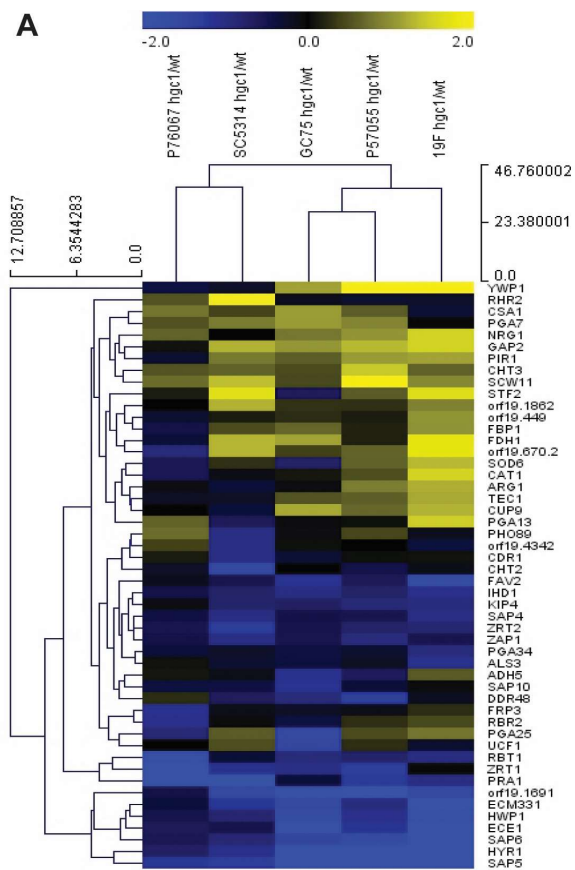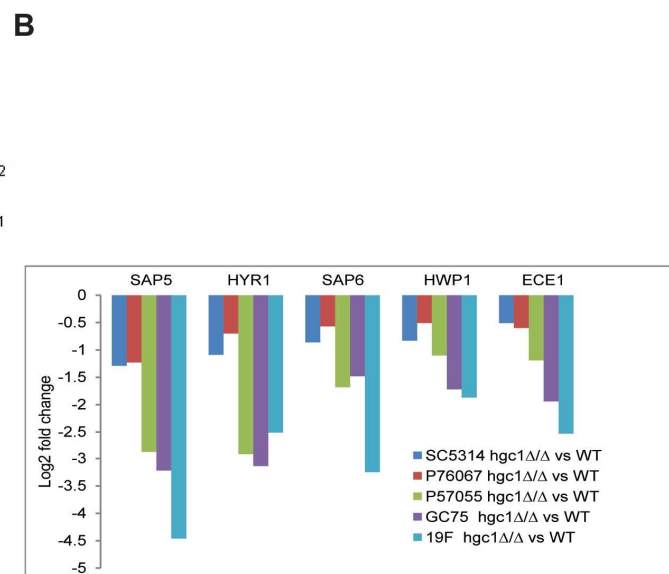

Supplement: jkad151_Supplementary_Data [file jkad151_supplementary_data.zip › Figure_S1_G3-2023-404262.pdf]
